# Calcium-mediated perception and defense responses activated in plant cells by metabolite mixtures secreted by the biocontrol fungus *Trichoderma atroviride*

**DOI:** 10.1186/1471-2229-7-41

**Published:** 2007-07-30

**Authors:** Lorella Navazio, Barbara Baldan, Roberto Moscatiello, Anna Zuppini, Sheridan L Woo, Paola Mariani, Matteo Lorito

**Affiliations:** 1 Dipartimento di Biologia, Università di Padova, Via U. Bassi 58/B, 35131 Padova, Italy; 2 Dipartimento di Arboricoltura, Botanica e Patologia Vegetale, Università di Napoli "Federico II", Via Università 100, 80055 Portici (NA), Italy

## Abstract

**Background:**

Calcium is commonly involved as intracellular messenger in the transduction by plants of a wide range of biotic stimuli, including signals from pathogenic and symbiotic fungi. *Trichoderma *spp. are largely used in the biological control of plant diseases caused by fungal phytopathogens and are able to colonize plant roots. Early molecular events underlying their association with plants are relatively unknown.

**Results:**

Here, we investigated the effects on plant cells of metabolite complexes secreted by *Trichoderma atroviride *wild type P1 and a deletion mutant of this strain on the level of cytosolic free Ca^2+ ^and activation of defense responses. *Trichoderma *culture filtrates were obtained by growing the fungus alone or in direct antagonism with its fungal host, the necrotrophic pathogen *Botrytis cinerea*, and then separated in two fractions (>3 and <3 kDa). When applied to aequorin-expressing soybean (*Glycine max *L.) cell suspension cultures, *Trichoderma *and *Botrytis *metabolite mixtures were distinctively perceived and activated transient intracellular Ca^2+ ^elevations with different kinetics, specific patterns of intracellular accumulation of reactive oxygen species and induction of cell death. Both Ca^2+ ^signature and cellular effects were modified by the culture medium from the knock-out mutant of *Trichoderma*, defective for the production of the secreted 42 kDa endochitinase.

**Conclusion:**

New insights are provided into the mechanism of interaction between *Trichoderma *and plants, indicating that secreted fungal molecules are sensed by plant cells through intracellular Ca^2+ ^changes. Plant cells are able to discriminate signals originating in the single or two-fungal partner interaction and modulate defense responses.

## Background

*Trichoderma *spp. are ubiquitous free-living soil fungi which act as biocontrol agents against several fungal phytopathogens. They are commercially applied as biopesticides, thus limiting the abuse of chemical fungicides [1,2]. The antagonist activity of *Trichoderma *depends on multiple synergistic mechanisms, including a direct interaction with the pathogenic partner (mycoparasitism), as well as indirect mechanisms based on competition for space and nutrients [3,4]. *Trichoderma *strains are rhizosphere competent, *i.e*. able to grow in association with plant roots, and can actually penetrate the first few layers of plant tissues [5,6]. The effects of *Trichoderma *colonization on plants include an improvement of plant growth and metabolism, as well as the induction of systemic and localized resistance to phytopathogenic fungi, bacteria and viruses (reviewed by [4]). Even though the physiological changes concerning the plant as a whole and induced by *Trichoderma *spp. have been relatively well investigated, there are only few reports on the mechanisms through which plant cells perceive fungal metabolites secreted during biocontrol. These fungal molecules, which include proteins, peptides, oligosaccharides and antibiotics, act naturally in mixtures. The presence in the *Trichoderma *exudates of many classes of chemical components potentially acting as elicitors may explain the ability of this fungus to activate induced systemic resistance (ISR) virtually on any plant variety [7].

During plant-fungal interactions an extensive exchange of molecular messages occurs. Variation in cytosolic free Ca^2+ ^concentration ([Ca^2+^]_cyt_) is a well-known early component of signal transduction pathways involved in plant-pathogen interactions [8,9]. Plants respond to pathogen attack through a rapidly induced [Ca^2+^]_cyt _elevation, which in turn initiates a cascade of reactions leading to activation of defense responses. No information is still available on the possible involvement of Ca^2+ ^as second messenger in the mechanism of *Trichoderma *perception by plants.

In this paper we investigated plant cell responses, including intracellular Ca^2+ ^variations, to *Trichoderma *metabolites released in the culture media of the fungus grown alone or in direct antagonism with a *Botrytis cinerea *strain susceptible to mycoparasitic attack by *T. atroviride *P1. In addition, we compared the effect of metabolite mixtures from both *T. atroviride *strain P1 wild type and a knock-out mutant of it, defective in the production of an endochitinase found to be important for biocontrol [10]. Our results indicate that plant cells are able to selectively perceive through Ca^2+ ^messages macromolecule components of the fungal culture filtrates, released in the different experimental conditions. Specific [Ca^2+^]_cyt _changes and levels of intracellular accumulation of reactive oxygen species (ROS), reduction in cell viability and occurrence of programmed cell death (PCD)/necrosis were detected.

## Results

### *Trichoderma *metabolite mixtures activate a Ca^2+^-mediated signalling in soybean cells

**Figure 1:**
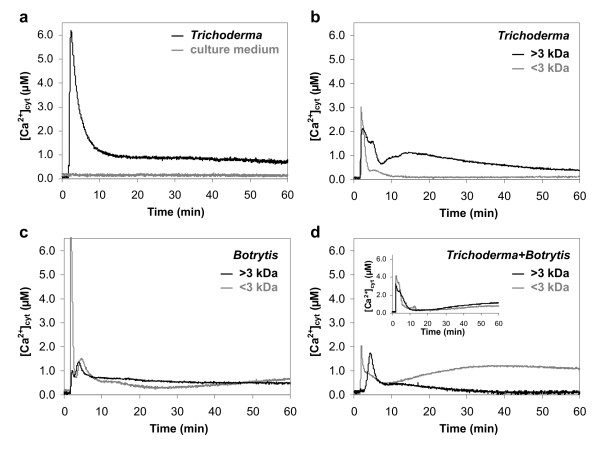
**Monitoring of [Ca^2+^]_cyt _in soybean cells challenged with fungal metabolite mixtures**. Cells were treated with: the whole culture filtrate of *Trichoderma *(*black trace*) or non-inoculated culture medium (*grey trace*) (a); >3 kDa (*black trace*) and <3 kDa (*grey trace*) fractions from culture filtrates of *Trichoderma *(b), *Botrytis *(c), and *Trichoderma *grown in the presence of *Botrytis *(d). In c, the first peak of the Ca^2+ ^transient induced by the >3 kDa metabolites is represented out of scale. In d, the inset shows the [Ca^2+^]_cyt _changes induced by the simultaneous application of the metabolite fractions (>3 kDa, *black trace*; <3 kDa, *grey trace*) from separately grown *Trichoderma *and *Botrytis*. Fungal filtrates were applied to cells after 100 s. These and the following traces have been chosen to best represent the mean results from at least three repetitions.

Fungal culture filtrates obtained from *T. atroviride *strain P1 wild type were tested on soybean cells stably expressing in the cytosol the bioluminescent Ca^2+ ^indicator aequorin. In the Ca^2+ ^measurement experiments fungal metabolite mixtures were applied to cells at a dose (4-fold concentrated culture medium) corresponding to that commonly used for *in vivo *bioassays of physiological effects (*i.e*. ISR and elicitor activity) on plants [10]. In preliminary dose-response experiments, the above concentration was found to induce about half of the maximum effect on [Ca^2+^]_cyt _increase (data not shown). The whole culture filtrate of *Trichoderma *elicited a strong Ca^2+ ^elevation that was generated without an evident lag phase after the metabolite mixture application. A *Trichoderma *"Ca^2+ ^signature" could be identified, which was characterized by a maximum of [Ca^2+^]_cyt _(6.09 ± 0.11 μM), reached after about 1 min, followed by a decrease within 20 min to 0.75 ± 0.06 μM, without returning to resting values (~100 nM) (Fig. 1a). No [Ca^2+^]_cyt _change was observed in control cells treated with the non-inoculated fungal culture medium (Fig. 1a). The *Trichoderma *metabolites were fractionated by using a 3 kDa cut-off and the two separated fractions were applied to soybean cells. The resulting Ca^2+ ^transients showed, after a first Ca^2+ ^peak nearly superimposable in time, very different kinetic trends characterized by a slow and modulated pattern of signal dissipation with the >3 kDa fraction, and a rapid decline of the Ca^2+ ^concentration to the basal level with the <3 kDa one (Fig. 1b). The combination of these two Ca^2+ ^traces plus a plausible synergistic effect of the molecular components of the two fractions  may account for the kinetics of the Ca^2+ ^change observed with the unfractionated metabolite mixture (Fig. 1a).

In order to determine whether the *Trichoderma *Ca^2+ ^signature is modified when the fungus is cultured with the pathogen *B. cinerea*, we tested metabolite mixtures produced by *B. cinerea *grown alone and during the coculture of these two fungi. Size-fractionated culture filtrates from the pathogenic fungus triggered in soybean cells Ca^2+ ^changes characterized by special features, such as an exceptionally high Ca^2+ ^elevation (7.53 ± 0.15 μM) caused by the <3 kDa metabolites, and a final long-lasting sustained Ca^2+ ^level recorded with both <3 kDa (0.66 ± 0.04 μM) and >3 kDa (0.47 ± 0.03 μM) fractions (Fig. 1c). The Ca^2+ ^transients observed upon cell treatment with both the fractions derived from *Trichoderma *cultured in the presence of *Botrytis *showed a single main Ca^2+ ^peak occurring at different time values and, with the <3 kDa fraction, the persistence of a very high sustained plateau (about 9-fold higher than the basal level) (Fig. 1d). It is noteworthy that different kinetics of the Ca^2+ ^signals were generated in soybean cells by the co-application of the filtrates (both >3 and <3 kDa) from the two separately-grown fungi (Fig. 1d, inset) in comparison to the traces induced by those of the cocultured fungi (Fig. 1d). These results suggest that the antagonism condition modifies the quality/quantity of the molecules accumulated in the culture media and indicate that the presence of the phytopathogenic host may significantly affect the Ca^2+ ^response of plant cells to *Trichoderma*.

### The lack of a *Trichoderma *specific endochitinase modifies the kinetics of the Ca^2+ ^changes

**Figure 2:**
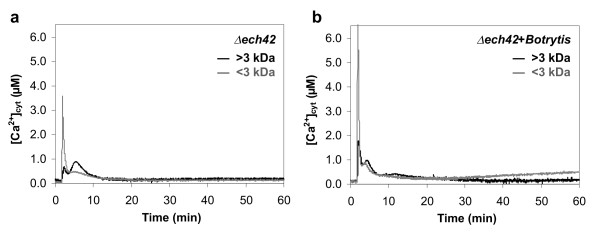
**[Ca^2+^]_cyt _responses of soybean cells to metabolite mixtures secreted by the *Trichoderma Δech42 *mutant**. Cells were treated with >3 kDa (*black trace*) or <3 kDa (*grey trace*) fractions of the metabolite mixtures secreted by the *Δech42 *mutant, grown alone (a) or in the presence of *Botrytis *(b).

The >3 kDa metabolite mixture from an endochitinase gene knock-out *Trichoderma *mutant, unable to produce the 42 kDa endochitinase (CHIT42) [10], induced a Ca^2+ ^transient clearly different from that of the wild type, both in the occurrence and level of the peaks. In addition, the Ca^2+ ^signal dissipated almost completely within 10 min (Fig. 2a, compare with Fig. 1b). These findings suggest that CHIT42 is among the *Trichoderma *metabolites that may be perceived as elicitor by plant cells, and is likely to account for the sustained [Ca^2+^]_cyt _level over the time. The <3 kDa mixture produced by the *Trichoderma Δech42 *mutant grown alone induced a Ca^2+ ^trace that did not significantly differ from the wild type (Fig. 2a, compared with Fig. 1b). On the other hand, when the *Δech42 *mutant was cocultivated with *B. cinerea*, also the <3 kDa metabolite mixture generated a Ca^2+ ^profile quite different from the corresponding wild type fraction and more closely resembling the *Botrytis*-induced Ca^2+ ^change (Fig. 2b, compared with Fig. 1c, d).

### *Trichoderma *metabolite mixtures elicit defense reactions in plant cells

#### Intracellular ROS accumulation

**Figure 3:**
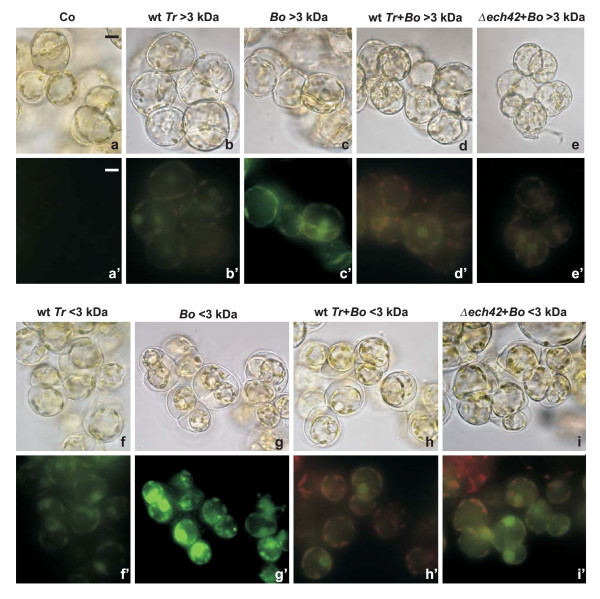
**Detection of intracellular ROS accumulation in soybean cells treated with fungal culture filtrates**. Intracellular ROS were detected by H_2_DCF-DA staining in control cells (Co, a'), in cells treated with >3 kDa (b'-e') and <3 kDa (f'-i') fractions of wild type *Trichoderma *(wt *Tr*, b'and f'), *Botrytis *(*Bo*, c'and g'), the coculture of wild type *Trichoderma *and *Botrytis *(wt *Tr*+*Bo*, d' and h') and the coculture of *Trichoderma Δ ech42 *mutant and *Botrytis *(*Δ ech42*+*Bo*, e'and i'). For each treatment light (a-i) and fluorescence (a'-i') microscope images of the same field are presented. All images were acquired with the same exposition time gauged to the higher fluorescence emission intensity obtained with the *Botrytis *<3 kDa fraction. Pictures represent typical examples after 10 min treatment. Bar: 10 μm.

One of the earliest plant responses at the cellular level to fungal pathogen infection is an increased production of intracellular ROS [11]. Preliminary tests indicated that a time interval between 5 and 10 min after the treatment was optimal to measure intracellular ROS accumulation by using dichlorofluorescein diacetate (DCF) [12] (data not shown). Compared to control cells, that showed no fluorescence at all (Fig 3a'), both >3 and <3 kDa *Trichoderma *metabolite fractions induced a faint detectable signal (Fig. 3b' and 3f'). As expected in the case of a necrotrophic pathogen, *Botrytis *filtrates, mainly <3 kDa, induced a level of fluorescence markedly higher (Fig. 3c' and 3g') than that of the biocontrol agent. ROS accumulation was very low when metabolites from *Trichoderma *cocultured with *Botrytis *were applied (Fig. 3d' and 3h'). In particular, in the case of the <3 kDa *Trichoderma*+*Botrytis *fraction (Fig. 3h') the significant reduction in DCF fluorescence may be attributed to the high percentage of dead cells (60.4 ± 1.8 % after 10 min) (see also below). No evident differences were found when cells were treated with filtrates of the *Δech42 *mutant compared to the wild type (see for example Fig. 3e'), unless the <3 kDa fraction of *Δech42 *+ *Botrytis *was applied (Fig. 3i', compared with 3h'). These findings indicate that, besides the generation of specific Ca^2+ ^signatures, other processes are differentially affected by metabolites secreted by the phytopathogen and the biocontrol agent. It can be speculated that the induction of ROS does not play a major role in the plant cell response to *Trichoderma *metabolite mixtures.

#### Reduction in cell viability

**Figure 4:**
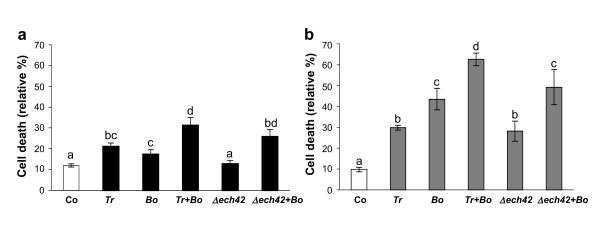
**Changes in cell viability in response to fungal culture filtrates**. Exponentially growing cells were incubated with >3 kDa (a, *black boxes*) and <3 kDa (b, *grey boxes*) fractions of the secreted fungal metabolite mixtures. Control cells (Co, *white boxes*) were incubated with culture medium only. All the abbreviations used for the treatments (wt *Tr*, *Bo*, *Δ ech42*) are as in Fig. 3. The 100% value correspond to cells treated for 10 min at 100°C. Data are means ± SD of three independent experiments. Bars labeled with a different letter differ significantly (*P *< 0.05) by Student's *t *test.

Intracellular Ca^2+ ^overload may determine cytotoxicity and cause either apoptotic or necrotic cell death [13]. In view of the high levels of [Ca^2+^]_cyt _induced by some of the fungal culture filtrates, their effect on cell viability was determined. Based on Evans Blue staining, all metabolite mixtures significantly increased after 30 min the percentage of dead cells in comparison with untreated controls, except the >3 kDa culture filtrate from the *Δech42 *mutant (Fig. 4). The reduction in cell viability was more remarkable with <3 kDa mixtures (Fig. 4b) than >3 kDa (Fig. 4a), suggesting a major toxic effect played by low MW metabolites.

#### Induction of programmed cell death

**Figure 5:**
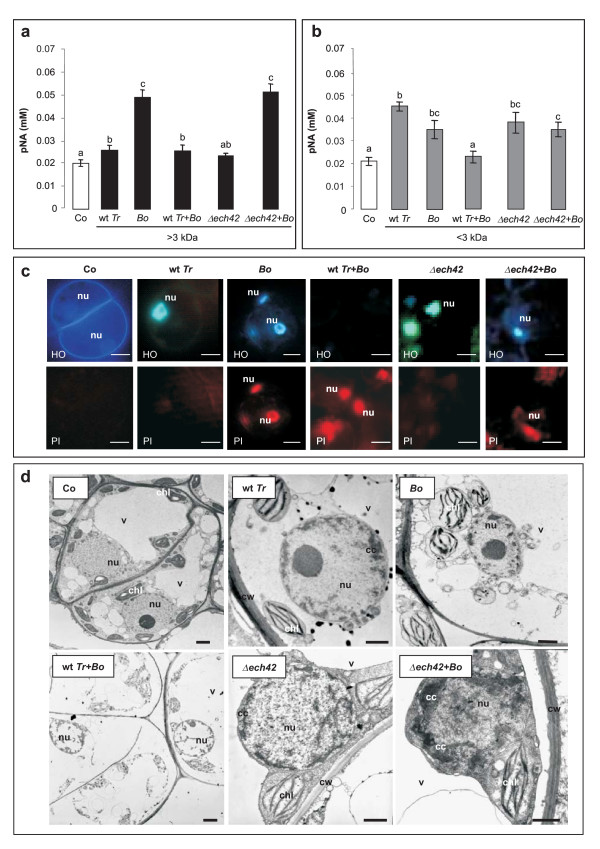
**Effect of the fungal metabolite mixtures on caspase 3-like activity, chromatin condensation and ultrastructure of soybean cells**. Panel a and b: caspase 3-like activity in cells treated for 30 min with >3 kDa (a, *black boxes*) and <3 kDa (b, *grey boxes*) fractions of the metabolite mixtures. Control cells (Co, *white boxes*) were incubated with culture medium only. All the abbreviations used for the treatments (wt *Tr*, *Bo*, *Δech42*) are as in Fig. 3. Data are means ± SD of three independent experiments. Bars labeled with a different letter differ significantly (*P *< 0.05) by Student's *t *test. Panel c: cells were treated with <3 kDa culture filtrates, stained with HO and PI, and observed under a fluorescence microscope. Pictures represent typical examples. nu, nucleus. Bars: 5 μm. Panel d: ultrastructural observations of control cells and cells incubated for 15 min with <3 kDa fungal metabolites. cc, chromatin condensation, chl, chloroplast, cw, cell wall, nu, nucleus, v, vacuole. Bars: 1 μm.

Detection of caspase activation, a strictly PCD-related event ([14] and references herein), was used to determine whether cell death induced by the fungal metabolite mixtures occurred via PCD rather than a necrotic event. In soybean control cells a low level of caspase 3-like activity, measured by quantification of free p-nitroaniline (0.018 ± 0.002 mM pNA), and probably due to normal cell turnover, was detected (Fig. 5a). In agreement with the results of the cell viability test, a significant increase of caspase 3-like protease activity was caused by 30 min application of both >3 and <3 kDa metabolite mixtures obtained from *Trichoderma *wild type grown alone (Fig. 5a and 5b). This indicates that PCD is part of the plant cell response to *Trichoderma *metabolites. Interestingly, the <3 kDa fraction obtained from the *Trichoderma*-*Botrytis *coculture, although generating the maximal cell death percentage (Fig. 4b), was not found to trigger a significant caspase 3-like activation (Fig. 5b), suggesting the induction of a different mode of cell death.

When cells were treated with the corresponding fraction of the *Δech42 Trichoderma *mutant cocultured with *Botrytis*, a statistically significant activity of caspase 3-like protease was recorded, and this value (0.038 ± 0.004 mM pNA) approached that obtained with the <3 kDa *Botrytis *filtrate (0.034 ± 0.003 mM pNA). In all experiments, the addition of a caspase 3 specific inhibitor (Ac-DEVD-CHO) lowered the amount of free pNA released from the substrate to the level of the control (data not shown), thus confirming the validity of the test for caspase 3-like activity.

#### Chromatin condensation and morphological cell alterations

Hoechst 33342 (HO)/Propidium Iodide (PI) staining followed by morphological analysis provided additional information on the type of cell death caused by the fungal metabolite mixtures produced in all the considered experimental conditions. Fig. 5c shows the staining pattern of soybean cells incubated with the plasma membrane-permeable DNA-binding agent HO and with the impermeant dye PI in the presence or absence of the <3 kDa fraction of the different fungal mixtures. Control cells had a faint or not detectable HO staining, with no evidence of chromatin condensation, and nuclei that did not stain with PI, indicating integrity of the plasma membrane. Electron microscope observations validated the healthy state of the cells (Fig. 5d). Cells treated with filtrates from *Trichoderma *wild type grown alone showed the prevalence of HO positive/PI negative nuclei, indicative of early PCD-like stages, characterized by chromatin condensation and an intact plasma membrane (Fig. 5c). Ultrastructural analyses confirmed these findings, showing small lumps of condensed chromatin close to an intact nuclear envelope and plasma membrane stuck to the cell wall in about 70% of the cells (Fig. 5d).

Most cells incubated with <3 kDa *Botrytis *metabolite mixture were HO positive/PI positive (late PCD stages), with both condensed chromatin and a functionally altered plasma membrane (Fig. 5c). Electron microscope observations indicated an evident chromatin condensation just beneath the nuclear envelope, chloroplasts and mitochondria altered in their organization, and plasma membrane detached from the cell wall (Fig. 5d).

The HO negative/PI positive staining pattern obtained with the <3 kDa metabolite mixture secreted in the coculture medium of the two fungal strains (Fig. 5c) revealed the absence of chromatin condensation and the breakdown of the plasma membrane, both characteristics of a necrotic status of the cells. The induction of a necrotic pathway was also supported by the lack of caspase 3-like activation (Fig. 5b). In the majority of the cells, the ultrastructure appeared deeply affected, with the plasma membrane completely detached and nuclei with a disorganized or absent nucleoplasm and heterogeneous residual chromatin clumps. Chloroplasts and mitochondria looked remarkably altered in their structure (Fig. 5d). Cell necrosis was somehow expected since during antagonism/mycoparasitism *T. atroviride *strain P1 typically releases trichorzianines, <3 kDa secondary metabolites capable of directly killing cells by destructing the plasma membrane [15]. However, both the induction and effectiveness of these antibiotics require the action of endochitinases and other cell wall degrading enzymes on the host tissues, which explains the results obtained when the *ech42 *deletion mutant was used in the coculture instead of the wild type. In this case, the lack of the major chitinase activity may have reduced the induction and accumulation of the necrogenic metabolites, which resulted in a typical late PCD-like staining (HO positive/PI positive) (Fig. 5c). This is confirmed by electron microscope observations, which likewise suggest a change in the induced cell death pathway (necrosis *versus *PCD) when wild type *Trichoderma *is replaced by the mutant strain in the dual coculture (Fig. 5d). No ultrastructural differences were found between treatments with <3 kDa metabolites produced by either *Trichoderma *wild type or *Δech42 *mutant grown alone (Fig. 5d).

## Discussion and Conclusion

The aim of this study was to investigate how plants can sense the presence of a fungus able to effect plant disease control in the rhizosphere. As experimental system we used plant cell cultures which were challenged with *Trichoderma *metabolite mixtures. Moreover, possible modifications in the pattern of the secreted molecules were analysed by testing the effects of culture filtrates of *Trichoderma *growing in direct antagonism with the phytopathogenic fungus *Botrytis*. The use of suspension cultured plant cells as a simplified approach, although not closely mimicking the natural situation, offers several advantages in the dissection of complex cellular responses at a molecular level. These *in vitro *studies may represent a valuable starting point for future experiments to be carried out *in planta*.

The molecular nature of the elicitors produced by *Trichoderma *strains has been at least partially unravelled (see [3,4] for reviews). Some of these compounds have been tested in purity for their ability to induce expression of plant defense genes and disease resistance [16,7]. However, the most direct way to have an overview of the complex reactions and effects caused by the fungal metabolites in plants is to assay the natural mixtures. Plants have been found to be actually penetrated and colonized by the fungus at the root level [5,6], and thus the secreted fungal molecules directly interact with living plant cells. It is at the plant-fungus interface that the first steps of the molecular interaction occur and generate the multiple effects observed both *in vitro *and in agriculture conditions.

Our results highlight the induction of Ca^2+^-mediated signal perception as an early step during the interaction of soybean cells with *Trichoderma *metabolites. Although the involvement of Ca^2+ ^in pathogen sensing by plants has been frequently claimed [17], to our knowledge a Ca^2+^-mediated perception by plant cells of a fungal biocontrol agent has not yet been reported. Since Ca^2+ ^has been recently demonstrated to be involved also in the molecular communication between plant cells and mycorrhizal fungi [18], a transient variation in [Ca^2+^]_cyt _proves once again to be the most general way for plants to open a dialogue with their fungal partners. The specificity of the Ca^2+ ^changes that we recorded in the single and two-fungal partner interactions (pathogenic and antagonist fungus alone and in combination) guarantees that this intracellular messenger delivers to cells different messages, which are progressively decoded into definite downstream responses. A specificity of the perception mechanism by plant cells is confirmed by the fact that different patterns of intracellular ROS accumulation and cell death induction were determined by the application of the various fungal mixtures. This does not necessarily imply that the cascade of events leading to the physiological responses follows separate, independent pathways, but rather that a network of overlapping pathways may be activated [19,20]. In view of the complexity of signalling crosstalks, firm causal links among Ca^2+^, ROS and cell death are not easy to assess.

The fungal growth conditions used in this work are well-known to induce the accumulation in the *Trichoderma *culture filtrates of specific compounds including enzymes, oligosaccharides and secondary metabolites. Separation of fungal culture filtrates by a 3 kDa cut-off let us discriminate differential cell responses to the active molecules recovered in the two fractions.

The larger MW (>3 kDa) fraction is known to contain a battery of hydrolytic enzymes, released by *Trichoderma *[21-23] as well as *Botrytis *[24,25], capable of digesting the plant cell wall. Increasing evidence indicates that the elicitor function of fungal lytic enzymes is unrelated to their enzymatic activity, but instead due to the direct perception by plant cells of the protein *per se*, rather than just through their hydrolysis products [26-28].

The <3 kDa *Trichoderma *fraction includes, as major secondary metabolites, peptaibols such as trichorzianines A_1 _and B_1 _[15], which are known to form oligomeric voltage-dependent ion channels in the plasma membrane of fungal hosts and plant cells, thus affecting membrane permeability [29,30]; cell death may occur as a consequence of cytoplasmic leakage through these ion channels [31]. Oligosaccharides, gradually released by the action of *Trichoderma *hydrolytic enzymes on the fungal host cell wall, may also be active components of the small MW fraction generated in the two-way interaction (*Trichoderma*-*Botrytis*). They are perceived by both the biocontrol agent as mycoparasitism/antagonism inducers [32] and by plant cells as elicitors [33,7]. Chitooligomers have been demonstrated to activate in plant cells an increase in [Ca^2+^]_cyt _[34,35] and defense responses [33,36]. All the <3 kDa fractions tested were found to induce in plant cells effects on both Ca^2+ ^changes and physiological parameters more remarkable than the higher MW mixtures. The enhancement of the cellular responses that we recorded upon cell treatment with the *Trichoderma*-*Botrytis *coculture filtrates cannot be attributed to the mere co-presence of elicitors released by the two fungi alone. Instead, qualitative/quantitative differences in the secreted compound mixture may arise when the biocontrol agent and the pathogen are grown together in direct antagonism, due to competition for nutrients and/or a direct effect of mycoparasitism [7,38]. Isolates of *T. harzianum *have been previously shown to reduce the activity of hydrolytic enzymes produced by *B. cinerea *[37]. Furthermore, the activity of *Trichoderma *hydrolytic enzymes gradually releases oligosaccharides from the *Botrytis *cell wall, which accumulate in the coculture medium. Our results indicate that plant cells are able to sense different elicitors that *Trichoderma *mainly addresses to its fungal host and consequently activate their own signal transduction pathway. This notion is also supported by recent findings concerning the activation in plants of a specific pattern of gene expression by the same *Trichoderma *strain [38].

Reduction in cell viability was recorded with all the metabolite mixtures of either *Trichoderma *or *Botrytis*. This result is expected for the pathogen, for which a Ca^2+^- and caspase-mediated hypersensitive response (HR) has already been reported [39-41], but also not surprising for the biocontrol agent. It is a common finding that plant roots and seeds treated with *Trichoderma *are dotted with small necrotic spots probably caused by a HR, which results in localized callose deposition that limits the colonization of plant tissues by the fungus to the first few layers of cells [5,3].

The lack of the 42 kDa endochitinase in the high MW fraction of the deletion mutant culture medium [10] determined a Ca^2+ ^transient clearly different from that of the wild type. This result indicates that the knock-out of the *ech42 *gene and its effect on the molecules secreted in the culture medium deeply modifies the fungal signal which is perceived by plant cells through Ca^2+^. Furthermore, the inactivation of the *ech42 *gene seems to produce, during the two-way interaction (*Δech42*-*Botrytis*), a <3 kDa metabolite mixture less necrogenic (in terms of ROS accumulation, activation of caspase 3-like protease and mode of cell death) than in wild type *Trichoderma*-*Botrytis*. This fraction is also able to induce a PCD pathway instead of a necrotic cell death, probably because of a reduced secretion of toxic secondary metabolites by the biocontrol agent. It has been demonstrated that the knock-out of the 42 kDa endochitinase alters the biocontrol ability of *Trichoderma virens *[42] and reduces the mycoparasitic and disease control efficacy *in vivo *of *T. atroviride *strain P1 [10]. In addition, the ISR-inducing ability of the *Δech42 *mutant is also lower than the wild type in assays where bean roots treated with *Trichoderma *are leaf-inoculated with *B. cinerea *(M. Lorito, unpublished). It has been previously demonstrated that complementation of the mutant culture filtrate with the purified 42 kDa endochitinase fully recovers the *Trichoderma *biocontrol activity [10]. Application to plant cells of the unfractionated *Trichoderma *culture filtrate complemented with CHIT 42 may provide a useful validation of the results obtained in this paper. Nevertheless, in view of possible pleiotropic effects of the endochitinase deletion on the fungal metabolism, this experimental approach may lead to an oversimplification of the complex network of synergistic interactions between the *Trichoderma *bioactive molecules [43].

The changes in [Ca^2+^]_cyt _triggered by *Trichoderma *metabolites in plant cells, although monitored only *in vitro*, provide new insight into the mechanisms by which these beneficial fungi affect plant physiology and resistance to stress. Our findings suggest the chance of using the *Trichoderma *secreted molecules, in mixtures or purified, as elicitor treatments against phytopathogens. This possibility is particularly intriguing, since a recognition of the biocontrol agent metabolites would allow the plant to perceive the presence of *Trichoderma*, thus pre-activating defense mechanisms against different pathogens [44], and also inducing a variety of other beneficial effects (*i.e*. promotion of plant growth, nutrient uptake, seed germination, resistance to abiotic stresses).

## Methods

### Fungal strains, growth conditions and preparation of culture filtrates

The wild type *Trichoderma atroviride *strain P1 [45] and its *ech42 *gene (encoding CHIT42 endochitinase) disruption mutant [10] were maintained at 25°C on potato dextrose agar (PDA) and as spore suspension in 10% glycerol at -40°C. The *Botrytis cinerea *strain 319 was isolated from tobacco, grown at 25°C on malt extract agar and kept as a spore suspension in 10% glycerol at -40°C. Fungal starter cultures were obtained in potato dextrose broth at 25°C, 150 rpm for 3 days with light, collected by centrifugation, rinsed with sterile distilled water, and used to inoculate a salt medium [46] containing 0.1 % (w/v) sucrose and 0.1 % (w/v) peptone. The cultures were grown at 25°C, 150 rpm, with light, for 3 days. Culture filtrates and the substrate alone, used as a control, were filter sterilized (0.22 μm), concentrated by roto-evaporation approximately 20-fold and fractionated with YM-3 MW (3000 Da cut-off) (Amicon Centriprep, Millipore) at 4000 rpm 6°C. The samples used as metabolite mixtures were: the whole concentrated filtrate, the fraction >3000 Da and that <3000 Da for each single fungus (*Trichoderma*, *Δech42 *mutant, *Botrytis*), plus the extracts of P1, or the *Δech42 *mutant, grown in the presence of *Botrytis*. For plant cell treatments, fungal culture filtrates were lyophilized and resuspended in plant cell culture medium. The final dose applied to cells corresponded to 4-fold concentrated fungal medium.

### Plant cell cultures

Cell suspension cultures of soybean (*Glycine max *L., line 6.6.12) stably expressing cytosolic aequorin were maintained as described by [34]. Cell treatments with fungal culture filtrates were performed two weeks after reinoculation, during the exponential growth phase of the cells.

### Aequorin-dependent Ca^2+ ^measurements

*In vivo *reconstitution of aequorin and Ca^2+ ^measurements were carried out as previously described [18].

### Intracellular ROS detection

Intracellular production of reactive oxygen species (ROS) was measured according to [12], by loading the cells with 15 μM 2',7'- dichlorodihydrofluorescein diacetate (H_2_DCF-DA, Molecular Probes, Leiden, The Netherlands). This non polar compound is actively taken up by cells and converted by esterases in H_2_DCF, a non fluorescent molecule, which is rapidly oxidized to the highly fluorescent DCF by intracellular peroxides. Treatments with fungal culture filtrates were carried out 10 min after dye loading and extensive washing. DCF was excited at 488 nm and emitted fluorescence was detected through a 520 bandpass filter. Cells were observed within 10 min.

### Cell viability

Cell viability was determined, after 30 min treatment with the fungal culture filtrates, by the Evans Blue method [47].

### Caspase 3-like activity

Caspase 3-like activity was measured, after 30 min cell treatment, using the "caspase-3 colorimetric activity assay kit" (Chemicon International, Inc., Temecula, CA), as previously described [26] by quantification of free p-nitroaniline (pNA) released by the enzymatic cleavage of the caspase 3 synthetic substrate Ac-DEVD-pNA.

### Hoechst 33342 (HO) and Propidium Iodide (PI) staining

After 30 min treatment with the different fungal culture filtrates, cells were incubated for 10 min in darkness with 8 μg/ml HO and 5 μg/ml PI (Sigma-Aldrich, St. Louis, USA) at room temperature. Cells were observed using a fluorescence microscope with an excitation light of 350 nm and 570 nm for HO and PI, respectively.

### Transmission electron microscopy

Cells were collected after 15 min treatment and processed as previously described [48].

### Statistical analysis

Data were expressed as mean ± S.D. The statistical significance of differences (*P *< 0.05) between means was evaluated using Student's *t *test.

## Authors' contributions

LN carried out Ca^2+ ^measurement assays and participated in drafting and editing the manuscript. BB carried out ROS detection, viability assays and electron microscope observations. RM maintained plant cell cultures and participated in the Ca^2+ ^measurement assays. AZ carried out the HO/PI test and caspase-3 like activity analysis. SW maintained fungal cultures, prepared and fractionated fungal metabolite mixtures and revised English language of the manuscript. PM participated in the design and coordination of the study, drafted the manuscript and participated in its editing. ML conceived of the study and helped to draft and edit the manuscript. All authors read and approved the final manuscript.
